# Relationships between lung function decline and skeletal muscle and fat mass changes: a longitudinal study in healthy individuals

**DOI:** 10.1002/jcsm.12821

**Published:** 2021-10-05

**Authors:** Han‐Ki Park, So‐Hee Lee, Suh‐Young Lee, Sun‐Sin Kim, Heung‐Woo Park

**Affiliations:** ^1^ Department of Internal Medicine, School of Medicine Kyungpook National University Daegu Korea; ^2^ Department of Internal Medicine Kyungpook National University Chilgok Hospital Daegu Korea; ^3^ Seoul National University Hospital Healthcare System Gangnam Center Seoul Korea; ^4^ Department of Internal Medicine Seoul National University Hospital Seoul Korea; ^5^ Institute of Allergy and Clinical Immunology Seoul National University Medical Research Center Seoul Korea; ^6^ Department of Internal Medicine Seoul National University College of Medicine Seoul Korea

**Keywords:** Lung function, FEV1, Body composition, Sarcopenia, Obesity, Health screening

## Abstract

**Background:**

The associations between long‐term changes in body mass composition and decline in lung function in healthy adults are unknown.

**Methods:**

Using a well‐defined health check‐up database, we first assessed individual longitudinal changes in muscle mass (MM) and fat mass (FM) measured via bioelectrical impedance analyses. Then we classified the enrolled individuals into five body composition groups according to their MM index (MMI) [MM (kg)/height (m)^2^] or FM index (FMI) [FM (kg)/height (m)^2^] change rate quartiles. Linear mixed models adjusted for age, smoking status, height, and body mass index were used to analyse the rate of forced expiratory volume in 1 s (FEV1) decline and body composition groups.

**Results:**

A total of 15 476 middle‐aged individuals (6088 women [mean age ± standard deviation: 50.74 ± 7.44] and 9388 men [mean age ± standard deviation: 49.36 ± 6.99]) were enrolled. The mean number of measurements was 6.96 (interquartile range [IQR]: 5–9) over an average follow‐up period of 8.95 years (IQR: 6.73–11.10). Decrease in MMI was significantly associated with accelerated FEV1 decline in men only (*P* = 1.7 × 10^−9^), while increase in FMI was significantly associated with accelerated FEV1 decline in both women and men (*P* = 7.9 × 10^−10^ and *P* < 2.0 × 10^−16^ respectively). Linear mixed model analyses indicated that annual increase of 0.1 kg/m^2^ in MMI was related to accelerated FEV1 decline by 30.79 mL/year (95% confidence interval [CI]: 26.10 to 35.48 mL/year) in men. Annual increase of 0.1 kg/m^2^ in FMI was related to accelerated FEV1 decline by 59.65 mL/year in men (95% CI: 56.84 to 62.28 mL/year) and by 22.84 mL/year in women (95% CI: 18.95 to 26.74 mL/year). In body composition analysis, we found increase in MMI was significantly associated with attenuated FEV1 decline in men only (*P* = 1.7 × 10^−9^), while increase in FMI was significantly associated with accelerated FEV1 decline in both women and men (*P* = 7.9 × 10^−10^ and *P* < 2.0 × 10^−16^ respectively). Individuals characterized with gain MM combined with loss of FM were associated with the most favourable outcome (i.e. the smallest rate of decline in FEV1) in both women and men. In men, loss of FM over time is more closely related with attenuated FEV1 decline than change in MM (gain or loss).

**Conclusions:**

Change in body composition over time can be used to identify healthy middle‐aged individuals at high risk for rapid FEV1 decline.

## Introduction

Obesity and loss of skeletal muscle mass (MM), known as sarcopenia, are associated with rapid decline in lung function.^1^, ^2^ Because reduced MM can be frequently masked by increased fat mass (FM) in adults, the effects of obesity and sarcopenia should be considered together.^3^ In addition, body muscle and fat compositions change over time,^4^ and thus, it is important to evaluate the effects of long‐term changes in MM and FM as well on decline in lung function.

Bioelectrical impedance analysis (BIA) is a non‐invasive technique for assessing body composition and is widely used in routine health check‐ups. Segmental BIA provides a measure of appendicular skeletal MM, which can be used as a proxy for skeletal MM.^5^ Compared with the reference methods, such as dual‐energy X‐ray absorptiometry, segmental BIA is a reliable method to measure skeletal MM even in older populations.^6^ To evaluate decline in lung function appropriately, long‐term observation with no missing variables is essential. Therefore, a serial database obtained from routine health check‐ups would be a good source of data, despite the potential risk for bias due to selection.

In this study, using a well‐defined health check‐up database, we first determined individual rate of changes in MM and FM over a long period and classified them into five body composition groups according to their patterns of change. Then we examined whether long‐term changes in MM and FM would be associated with decline in lung function, as assessed via forced expiratory volume in 1 s (FEV1) in healthy individuals.

## Materials and Methods

### Study populations

The medical records of individuals who underwent health check‐ups at the Seoul National University Hospital Healthcare System Gangnam Center between October 2004 and May 2019 were reviewed. Collected data included age, gender, height, smoking status, co‐morbidities, and results of lung function tests. They were self‐recruited through routine health check‐ups, which were usually provided annually. Individuals who underwent at least three health check‐ups with a follow‐up period (between the first and last check‐ups) > 5 years were included. We excluded individuals who self‐reported any respiratory disease or had suggestive abnormalities in chest X‐ray or chest computed tomography for respiratory diseases affecting lung function or decline in lung function (e.g. asthma, chronic obstructive disease [COPD], and pulmonary fibrosis). Smokers were defined as those with a smoking history of >10 pack‐years, which is a significant predictor of accelerated decline in FEV1.^7^ The study protocol was approved and the need for informed consent from participants was waived by the Institutional Review Board of Seoul National University Hospital (H‐1601‐080‐734).

### Measurements

Lung function tests were routinely performed in all individuals who underwent health check‐ups using a flow sensing type spirometer, (MasterScreen Pneumo; Viasys Respiratory Care, Inc., San Diego, CA, USA) in accordance with the American Thoracic Society's recommendations.^8^ Forced vital capacity (FVC) and FEV1 were measured, and the results are expressed as absolute (mL) and predicted values (%) calculated from a formula based on the Korean population.^9^ All measurements were performed without bronchodilator inhalation.

Segmental single‐frequency BIA measurements were taken (Inbody 720; Biospace Corporation, Seoul, Korea) in individuals in their bare feet wearing light clothing. Segmental BIA measures the composition of limbs and trunk separately and provides the sum of predicted MM (kg) from the limbs as a proxy for skeletal MM and the whole‐body FM (kg). Both MM and FM were normalized for height by dividing by height (m)^2^, to generate the MM index (MMI) and FM index (FMI).

### Statistical analyses

As body composition differs markedly between women and men, analyses were performed separately for each gender. For each individual, the rate of decline in FEV1 (mL/year) and the rate of annual changes in MMI and FMI ([kg/m^2^]/year) were calculated using all measurements obtained over the follow‐up period. Simple correlations between the rate of FEV1 decline and the rate of changes in MMI and FMI were evaluated. We then classified individuals into quartile (Q1 [the lowest] to Q4 [the highest]) according to the rate of changes in MMI or FMI. To consider interactive changes between MM and FM over time, we divided individuals into five body composition groups based on their MMI and FMI change rate quartiles. Individuals belonging to Q1 and Q4 in each quartile were selected for body composition grouping to introduce the maximal difference: Group 1 (individuals belonging to both MMI change rate Q4 and FMI change rate Q1, representing ‘gain of MM and loss of FM’), Group 2 (individuals belonging to both MMI change rate Q4 and FMI change rate Q4, representing ‘gain of MM and prominent gain of FM’), Group 3 (individuals belonging to both MMI change rate Q1 and FMI change rate Q1, representing ‘prominent loss of MM and loss of FM’), Group 4 (individuals belonging to both MMI change rate Q1 and FMI change rate Q4 representing, ‘prominent loss of MM and prominent gain of FM’), and Group 5 (others, representing ‘control’).

Differences in FEV1 decline among groups were analysed using linear mixed models to identify predictors of body composition groups, with a fixed effect for year (duration of the follow‐up period) and a random effect for individuals to accommodate repeated measurements. These models included age, smoking status, height, and body mass index (BMI) at the first health check‐up as predictors. Statistical analyses were performed using R version 4.0.2 (R Foundation for Statistical Computing, Vienna, Austria), and a two‐sided *P* < 0.05 was taken to indicate statistical significance.

## Results

A total of 15 476 individuals were included in the analyses (6088 women and 9388 men). The mean number of FEV1 and segmental BIA measurements was 6.96 (interquartile range [IQR]: 5–9), and the mean observation period was 8.95 (IQR: 6.73–11.10) years.

**Figure 1:**
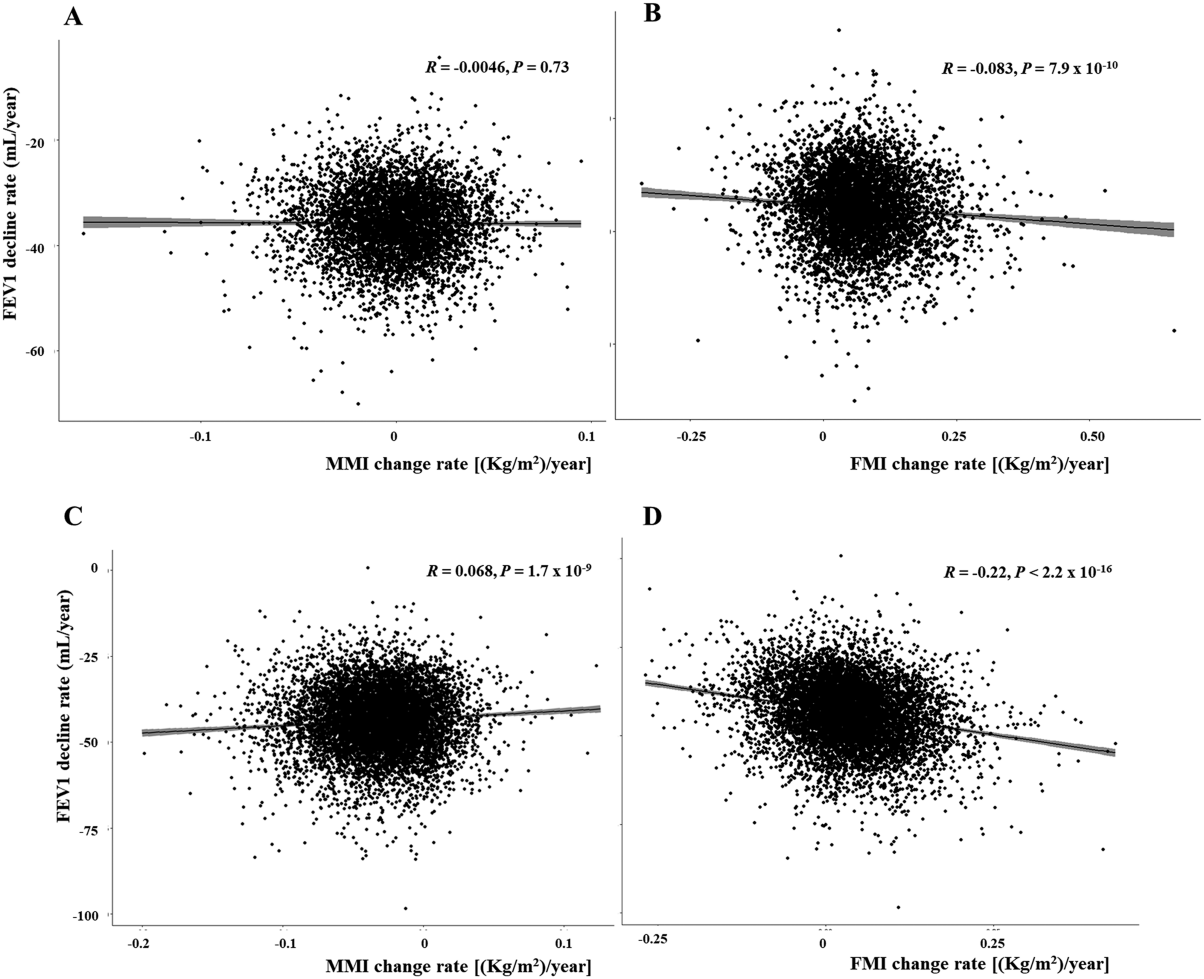
Correlations between a rate of decline in FEV1 and a rate of change in muscle or fat mass index. *(A)* Rate of decline in FEV1 vs. rate of change in MMI in women. *(B)* Rate of decline in FEV1 vs. rate of change in FMI in women. *(C)* Rate of decline in FEV1 vs. rate of change in MMI in men. *(D)* Rate of decline in FEV1 vs. rate of change in FMI in men. Correlation is measured by the Pearson's methods. FEV1, forced expiratory volume in one second, FMI; fat mass index (kg/m^2^); MMI; muscle mass index (kg/m^2^).

Consistent with previous reports, a rate of decline in FEV1 and a rate of change in body weight (kg/year) showed significant negative correlations (i.e. gain of body weight accelerated FEV1 decline) in both women and men significantly (Supporting Information, *Figure* S1). As expected, body weight change was positively correlated with change in MMI or FMI with significance, but FMI showed a stronger correlation than MMI in both women and men (*Figure* S2). *Figure* 1 shows correlations between the rate of decline in FEV1 and the rate of change in MMI or FMI in women and men. Increase in MMI was significantly associated with attenuated FEV1 decline in men only (*P* = 1.7 × 10^−9^), while increase in FMI was significantly associated with accelerated FEV1 decline in both women and men (*P* = 7.9 × 10^−10^ and *P* < 2.0 × 10^−16^ respectively). Linear mixed model analyses indicated that annual increase of 0.1 kg/m^2^ in MMI (equivalent to annual MM loss of 0.289 kg in an individual with a height of 1.7 m) was related to attenuated FEV1 decline by 30.79 mL/year (95% confidence interval [CI: 26.10 to 35.48 mL/year) in men. Annual increase of 0.1 kg/m^2^ in FMI (equivalent to annual FM gain of 0.289 kg in an individual with a height of 1.7 m) was related to accelerated FEV1 decline by 59.65 mL/year in men (95% CI: 56.84 to 62.28 mL/year) and by 22.84 mL/year in women (95% CI: 18.95 to 26.74 mL/year).

**Figure 2:**
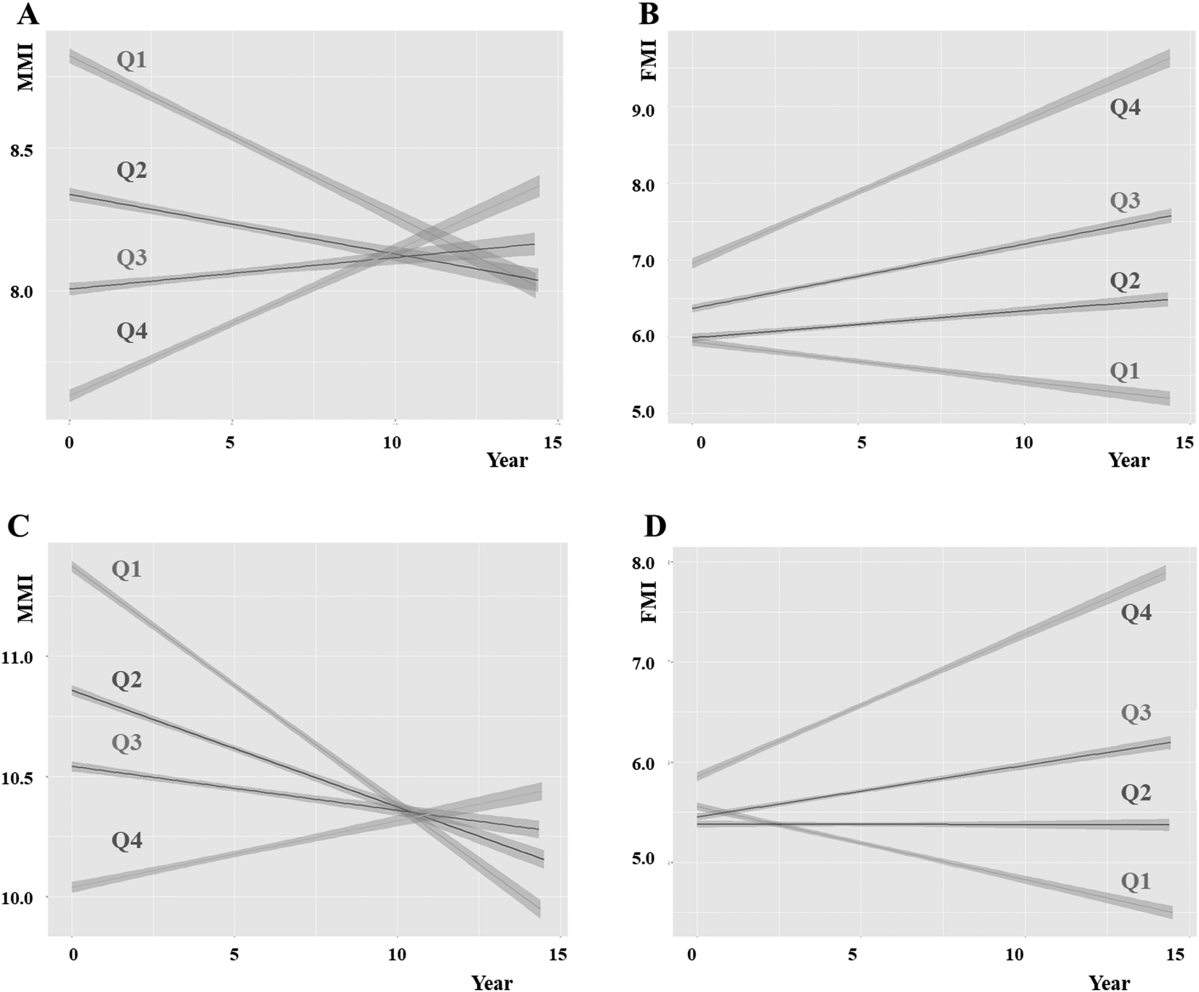
Changes in muscle and fat mass index over time according to quartiles. *(A)* MMI change in woman. *(B)* FMI change in woman. *(C)* MMI change in man. *(D)* FMI change in man. FMI, fat mass index (kg/m^2^); MMI, muscle mass index (kg/m^2^); Q, quartile.

**Figure 3:**
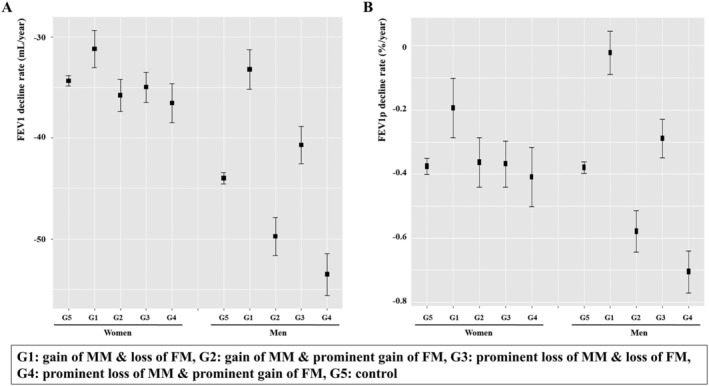
Rates of decline in FEV1 and FEV1p of five body composition groups in women and men. Data are presented as mean with 95% confidence interval. FEV1, forced expiratory volume in one second; FEV1p, FEV1 predicted value; MM, muscle mass; FM, fat mass; G, group.

*Figure* 2 shows changes in MMI and FMI over time according to quartiles in women and men. The distribution of MMI and FMI change rate quartiles is presented in *Table* S1. As mentioned earlier, the participants enrolled were classified into five body composition groups based on their MMI or FMI change rate quartiles. *Tables* 1 and 2 show baseline characteristics (measured at the first health check‐up) of the five body composition groups in women and men, respectively. The rate of decline in FEV1 according to body composition groups are presented in *Figure* 3 and *Table* 3. Using a linear mixed model, we found that only Group 1 characterized by gain of MM combined with loss of FM showed a significant association with attenuated FEV1 decline compared with Group 5 (control) in women (*P* = 0.045). Groups 1 and 3 characterized by loss of FM showed a significant association with attenuated FEV1 decline compared with Group 5 in men (*P* < 2.0 × 10^−16^ and *P* = 7.2 × 10^−3^ respectively). Interestingly, Groups 2 and 4 characterized by prominent gain of FM were associated significantly with accelerated FEV1 decline compared with Group 5 in men (*P* < 2.0 × 10^−16^ and *P* < 2.0 × 10^−16^, respectively). To confirm our observations, we performed the same analysis again using FEV1 predicted value (FEV1p). FEV1p is a relative value adjusted by age and height, whereas FEV1 is an absolute value. As shown in *Figure* 3 and *Table* 3, we obtained results similar to those of FEV1.

**Table 1:** Baseline characteristics (measured at the first health check‐up) of body composition groups in women

	Group 1 (*n* = 423)	*P* value^a^	Group 2 (*n* = 303)	*P* value^a^	Group 3 (*n* = 325)	*P* value^a^	Group 4 (*n* = 414)	*P* value^a^	Group 5 (*n* = 4623)
Age	48.33 (7.01)	4.3 × 10^−3^	47.95 (6.41)	7.1 × 10^−4^	49.22 (6.85)	NS	51.77 (7.59)	1.9 × 10^−11^	49.34 (6.92)
Follow up, year	9.10 (2.63)	NS	9.15 (2.91)	NS	9.36 (2.59)	4.4 × 10^−4^	9.17 (2.35)	0.014	8.85 (2.66)
Check‐up, *n*	7.07 (2.82)	4.4 × 10^−3^	6.89 (2.42)	NS	7.16 (3.05)	1.2 × 10^−3^	6.67 (2.70)	NS	6.67 (2.96)
Smoker, *n*	31 (7.32%)	NS	20 (6.60%)	NS	26 (8.00%)	0.024	29 (7.00%)	NS	236 (5.10%)
Height, cm	157.09 (5.14)	1.0 × 10^−7^	156.50 (4.84)	4.7 × 10^−11^	159.88 (5.17)	6.7 × 10^−7^	159.16 (4.65)	5.2 × 10^−3^	158.45 (4.96)
BMI, kg/m^2^	20.18 (1.86)	<2.0 × 10^−16^	21.39 (2.18)	6.6 × 10^−4^	23.60 (2.74)	<2.0 × 10^−16^	24.47 (3.09)	<2.0 × 10^−16^	21.87 (2.37)
FEV1, mL	2508.72 (386.13)	NS	2490.29 (368.99)	NS	2612.40 (394.73)	1.4 × 10^−4^	2492.02 (381.14)	NS	2529.24 (374.61)
FEV1p, %	106.54 (13.06)	NS	106.54 (13.58)	NS	107.09 (12.44)	NS	106.12 (13.11)	NS	106.13 (13.03)
FVC, mL	3001.06 (446.94)	0.010	2979.34 (429.43)	2.3 × 10^−3^	3171.50 (445.76)	3.1 × 10^−6^	3054.92 (435.73)	NS	3056.70 (424.88)
FVCp, %	97.03 (11.83)	NS	96.62 (11.80)	NS	98.88 (10.34)	0.027	98.11 (11.03)	NS	97.46 (11.25)
FEV1/FVC	0.83 (0.058)	2.56 × 10^−3^	0.83 (0.058)	0.010	0.82 (0.058)	NS	0.81 (0.056)	6.6 × 10^−5^	0.82 (0.058)
MMI, kg/m^2^	7.55 (0.60)	<2.0 × 10^−16^	7.73 (0.69)	<2.0 × 10^−16^	8.75 (0.74)	<2.0 × 10^−16^	8.93 (0.76)	<2.0 × 10^−16^	8.14 (0.71)
FMI, kg/m^2^	5.38 (1.35)	<2.0 × 10^−16^	6.29 (1.47)	NS	7.14 (1.96)	<2.0 × 10^−16^	7.71 (2.23)	<2.0 × 10^−16^	6.28 (1.65)
MMI change rate^b^	0.0029 (0.011)	<2.0 × 10^−16^	0.0030 (0.013)	<2.0 × 10^−16^	−0.033 (0.016)	<2.0 × 10^−16^	−0.034 (0.017)	<2.0 × 10^−16^	−0.00039 (0.019)
FMI change rate^b^	−0.024 (0.037)	<2.0 × 10^−16^	0.17 (0.059)	<2.0 × 10^−16^	−0.042 (0.055)	<2.0 × 10^−16^	0.17 (0.056)	<2.0 × 10^−16^	0.070 (0.065)

BMI, body mass index; FEV1, forced expiratory volume in one second; FEV1p, FEV1 predicted value; FMI, fat mass index (kg/m^2^); FVC, forced vital capacity; FVCp, FVC predicted value; MMI, muscle mass index (kg/m^2^); *n*, number; NS; not significant.

Data are presented as mean (standard deviation) except smoker (number [%]).

^a^ Compared with Group 5.

^b^ (kg/m^2^)/year.

**Table 2:** Baseline characteristics (measured at the first health check‐up) of body composition groups in men

	Group 1 (*n* = 542)	*P* value^a^	Group 2 (*n* = 449)	*P* value^a^	Group 3 (*n* = 562)	*P* value^a^	Group 4 (*n* = 654)	*P* value^a^	Group 5 (*n* = 7181)
Age	50.01 (7.38)	0.038	48.81 (6.84)	1.4 × 10^−7^	50.51 (7.06)	NS	53.35 (8.51)	<2.0 × 10^−16^	50.69 (7.33)
Follow up, year	9.16 (2.72)	5.1 × 10^−3^	9.13 (2.59)	0.021	9.29 (2.54)	4.3 × 10^−5^	9.23 (2.46)	2.0 × 10^−4^	8.84 (2.64)
Check‐up, *n*	7.62 (2.96)	1.9 × 10^−7^	7.28 (2.72)	0.020	7.57 (3.09)	9.2 × 10^−7^	7.43 (3.07)	5.5 × 10^−5^	6.96 (2.97)
Smoker, *n*	398 (73.43%)	NS	332 (73.94%)	NS	449 (79.89%)	4.1 × 10^−7^	483 (73.85%)	0.025	5003 (69.66%)
Height, cm	168.16 (5.36)	<2.0 × 10^−16^	169.20 (5.75)	2.7 × 10^−6^	172.04 (5.23)	5.5 × 10^−11^	171.98 (5.37)	1.9 × 10^−11^	170.46 (5.47)
BMI, kg/m^2^	23.12 (2.42)	0.043	23.05 (2.57)	NS	26.22 (2.42)	0.019	26.45 (2.63)	3.9 × 10^−3^	24.56 (16.69)
FEV1, mL	3368.43 (510.25)	NS	3426.34 (542.00)	NS	3436.54 (545.54)	NS	3303.04 (604.23)	9.1 × 10^−7^	3413.84 (542.20)
FEV1p, %	104.28 (12.89)	NS	103.54 (12.72)	NS	102.16 (12.55)	0.014	100.96 (14.10)	1.3 × 10^−6^	103.52 (12.73)
FVC, mL	4172.25 (592.44)	5.2 × 10^−4^	4249.24 (612.56)	NS	4325.58 (626.30)	0.031	4207.29 (681.26)	0.019	4267.10 (614.63)
FVCp, %	96.75 (11.04)	NS	96.53 (10.69)	NS	95.45 (10.64)	NS	94.27 (11.65)	9.2 × 10^−6^	96.26 (10.83)
FEV1/FVC	0.81 (0.061)	0.041	0.81 (0.067)	0.025	0.79 (0.062)	<2.0 × 10^−16^	0.78 (0.066)	2.2 × 10^−9^	0.80 (0.062)
MMI, kg/m^2^	10.04 (0.80)	<2.0 × 10^−16^	10.06 (0.93)	<2.0 × 10^−16^	11.35 (0.80)	<2.0 × 10^−16^	11.38 (0.95)	<2.0 × 10^−16^	10.63 (0.89)
FMI, kg/m^2^	5.06 (1.43)	3.2 × 10^−12^	5.00 (1.56)	1.6 × 10^−12^	6.35 (1.67)	<2.0 × 10^−16^	6.53 (1.73)	<2.0 × 10^−16^	5.50 (1.43)
MMI change rate^b^	0.0028 (0.015)	<2.0 × 10^−16^	0.0030 (0.019)	<2.0 × 10^−16^	−0.078 (0.020)	<2.0 × 10^−16^	−0.080 (0.021)	<2.0 × 10^−16^	−0.00035 (0.025)
FMI change rate^b^	−0.049 (0.034)	<2.0 × 10^−16^	0.12 (0.046)	<2.0 × 10^−16^	−0.062 (0.047)	<2.0 × 10^−16^	0.13 (0.046)	<2.0 × 10^−16^	0.034 (0.058)

BMI, body mass index; FEV1, forced expiratory volume in one second; FEV1p, FEV1 predicted value; FMI, fat mass index (kg/m^2^); FVC, forced vital capacity; FVCp, FVC predicted value; MMI, muscle mass index (kg/m^2^); *n*, number; NS; not significant.

Data are presented as mean (standard deviation) except smoker (number [%]).

^a^ Compared with Group 5.

^b^ (kg/m^2^)/year.

**Table 3:** Comparisons of rate of decline in FEV1 and FEV1p among body composition groups in women and men

Group	FEV1 decline rate (mL/year)			FEV1p decline rate (%/year)		
	Mean (95 CI)	*P* ^a^	*P* ^b^	Mean (95 CI)	*P* ^a^	*P* ^b^
Women						
Group 1	−31.20 (−33.03 to −29.36)	0.045	NA	−0.19 (−0.28 to −0.10)	4.0 × 10^−3^	NA
Group 2	−35.80 (−37.39 to −34.22)	NS	6.3 × 10^−3^	−0.36 (−0.44 to −0.28)	NS	0.050
Group 3	−34.98 (−36.50 to −33.49)	NS	0.048	−0.37 (−0.44 to −0.29)	NS	0.044
Group 4	−36.57 (−38.51 to −34.65)	NS	3.3 × 10^−4^	−0.41 (−0.50 to −0.31)	NS	1.5 × 10^−2^
Group 5	−34.37 (−34.88 to −33.86)	NA	0.045	−0.37 (−0.40 to −0.35)	NA	4.0 × 10^−3^
Men						
Group 1	−33.23 (−35.19 to −31.26)	<2.0 × 10^−16^	NA	−0.02 (−0.08 to 0.04)	<2.0 × 10^−16^	NA
Group 2	−49.75 (−51.65 to −47.88)	2.9 × 10^−7^	<2.0 × 10^−16^	−0.57 (−0.64 to −0.51)	4.4 × 10^−9^	<2.0 × 10^−16^
Group 3	−40.72 (−42.56 to −38.88)	7.2 × 10^−3^	1.1 × 10^−10^	−0.29 (−0.35 to −0.23)	1.1 × 10^−3^	2.7 × 10^−10^
Group 4	−53.52 (−55.61 to −51.46)	<2.0 × 10^−16^	<2.0 × 10^−16^	−0.70 (−0.77 to −0.64)	<2.0 × 10^−16^	<2.0 × 10^−16^
Group 5	−44.01 (−44.58 to −43.45)	NA	<2.0 × 10^−16^	−0.37 (−0.39 to −0.36)	NA	<2.0 × 10^−16^

95 CI, 95% confidence interval; FEV1, forced expiratory volume in one second; FEV1p, FEV1 predicted value; NA, not applicable; NS, not significant.

^a^ Compared with Group 5.

^b^ Compared with Group 1.

## Discussion

This study investigated the effects of longitudinal change in body composition derived from combinations of MMI and FMI change rates on FEV1 decline using a large health check‐up database. Specifically, we found that individuals characterized by gain of MM combined with loss of FM during follow‐up period are associated with the most favourable outcome, that is, the smallest decline in FEV1 in both women and men; in men, loss of FM over time is more closely associated with attenuated FEV1 decline than change in MM (gain or loss); and the associations of longitudinal change in body composition on FEV1 decline are more prominent in men than women.

There have been many reports of the possible associations between poor lung function and decreased skeletal MM (sarcopenia) or obesity measured using BMI.^10^, ^11^, ^12^, ^13^ However, most previous studies have evaluated the cross‐sectional association between sarcopenia and lung function, focusing on patients with COPD. Accordingly, there have few reports on the associations of sarcopenia with changes in lung function over time in healthy individuals. Body weight is a sum of MM and FM and thus does not precisely reflect body composition, although it can be measured easily in practice. We observed a significant correlation between a rate of change in body weight and a rate of decline in FEV1, consistent with a previous report.^13^ However, a rate of change in FMI showed a stronger association with a rate of change in body weight than that of MMI (*Figure* S2). Therefore, we could not evaluate the separate effects of change in MMI or FMI on decline in lung function, if focusing only on change in body weight. In addition, with aging, total body FM increases and concomitantly body lean mass and bone density decrease, which is thought to be independent from general and physiological fluctuations in weight and BMI.^14^ Consistent with these observations, sarcopenia with obesity is associated with higher levels of metabolic disorder and an increased risk for mortality than either obesity or sarcopenia alone.^15^ A cross‐sectional study showed that sarcopenic obesity was associated with lower FEV1 values than sarcopenia in man patients with COPD.^16^ By contrast, another cross‐sectional study performed in healthy elderly subjects did not observe different effects on FEV1 values between sarcopenic obesity and sarcopenia.^17^ Currently, there is no widely accepted definition of sarcopenic obesity, but it usually refers to a high BMI with sarcopenia measured via dual X‐ray absorptiometry.^18^ Taken together, our data using MMI or FMI instead of body weight or BMI provide new insight into the contributions of body composition‐related factors to the decline in lung function in healthy individuals. To the best of our knowledge, this is the first study to evaluate the effects of long‐term changes in MM and FM on the decline in lung function.

Loss of FM over time may be associated with attenuated FEV1 decline in men, regardless of change of MM. It means that even prominent loss of MM (i.e. Group 3 in this study) is associated with attenuated FEV1 decline in men unless it is combined with prominent gain of FM. This observation seems to contradict previous studies showing that sarcopenia is associated with poor lung function.^10^, ^11^, ^16^, ^17^ All previous studies were performed in patients with COPD or in healthy elderly populations, while the mean age of healthy individuals enrolled in this study was around 50, and they were followed up for an average of 9 years. This study also distinguished by its longitudinal nature. A recent study performed in young European adults (20–40 years) showed that moderate or high weight gain over 20 years was associated with accelerated decline in lung function, while weight loss was related to its attenuation.^13^ Interestingly, investigators in the UK reported that MMI declines in men from the age of 40, whereas in women, MMI is more stable and decreases only slightly among those in the higher percentiles.^19^ However, that study also found that FMI increases with age in both women and men.^19^

All these findings suggest that gain of body weight observed in middle‐aged men may be mainly due to gain of FM. Similarly, we found that gain of body weight over time is more closely related with gain of FM rather than MM in this study. Taken together, change in FM over time is a more important factor related to FEV1 decline in men in this age compared with elderly men. Additional studies for MM and FM changes over time, focusing on gender‐related or age‐related differences, are warranted.

Two potential mechanisms have been proposed to explain the associations between gain of FM and accelerated decline in lung function. The first is the mechanical effect on the lung. Abdominal and thoracic FM are likely to reduce vital capacity and to lead to expiratory flow limitation by limiting the room for lung expansion during inspiration.^20^ Second, gain of FM can impair lung function by inflammatory processes, as adipose tissue is a source of inflammatory mediators that can damage lung tissue and reduce airway diameter.^21^ For example, C‐reactive protein (CRP), an indicator of systemic inflammation in combination with high fat accumulation, is associated with poor lung function.^22^ Unfortunately, we could not gather information about chest compliance, but we could evaluate differences in mean serum levels of CRP among body composition groups, as CRP was measured at each health check‐up. Mean serum level of CRP in Group 1 was significantly lower compared with Group 5 in both women and men (*Table* 4). Meanwhile, mean serum level of CRP in Group 4 was significantly higher compared with Group 5 in men only. Systemic inflammation related to gain of FM over time may account for rapid FEV1 decline found in men.

**Table 4:** Levels of mean serum C‐reactive protein among body composition groups in women and men

	Group 1	*P* value^a^	Group 2	*P* value^a^	Group 3	*P* value^a^	Group 4	*P* value^a^	Group 5
Women CRP (mg/dL)	0.012 (0.16)	0.021	0.095 (0.13)	NS	0.077 (0.13)	NS	0.096 (0.14)	NS	0.096 (0.18)
Men CRP (mg/dL)	0.11 (0.14)	0.0013	0.14 (0.16)	NS	0.12 (0.14)	NS	0.17 (0.24)	0.0085	0.14 (0.25)

CRP, C‐reactive protein; NS, not significant.

Data are presented as mean (standard deviation).

^a^ Compared with Group 5.

Another interesting finding of this study was that the associations between body composition changes over time and FEV1 decline are more prominent in men than in women. After accounting for BMI, cross‐sectional analyses showed a strong inverse association between waist‐to‐hip ratio and FEV1 in men but not in women.^23^ A previous study showed that MMI decline was evident only in middle‐aged men, whereas FMI increased with age in both women and men in this age group.^19^ Moreover, a longitudinal population‐based study confirmed that the association between elevated CRP and FEV1 decline was only found in men.^24^ It is possible that sex hormone changes in these middle aged individuals are involved in this difference. Considering that the age at natural menopause IQR in Korean women was 47–52,^25^ most women enrolled in this study were in transitional state. Menopausal status was associated with accelerated lung function decline in women.^26^ However, the association was particularly prominent for FVC, and the adjusted mean FEV1 decline increased only by 3.8 mL/year in transitional women.^26^ In addition, a recent large study using UK biobank data showed that the effects of testosterone markers on FEV1 decline over the 8 year follow‐up were small in both women (mean age; 53.5) and men (mean age; 55.3).^27^ The differential effects of sex hormone on lung function decline need to be further evaluated. Taken together, these findings and our observations suggest that the associations between body composition changes and FEV1 decline are more prominent in men than in women, particularly in middle‐aged healthy individuals.

This study had several imitations. First, individuals included in the study underwent health check‐ups at only one centre, which may have introduced selection bias. Second, the health screening setting, itself, may be a source of selection bias (i.e. a predominance of middle‐aged men and smokers). These points should be taken into consideration when generalizing our observations to other populations. Finally, as post‐bronchodilator FEV1 was not measured, it was possible that individuals who potentially had asthma or COPD were included in the study population. However, individuals who reported respiratory diseases as co‐morbidities even at one health screening during the follow‐up period were excluded from the analyses, and 110 women (2.37%) and 581 men (8.09%) had an FEV1/FVC ratio < 0.7 at the first health check‐up. Therefore, the possibility of inaccuracies due to the inadvertent inclusion of asthma or COPD patients was low.

In conclusion, we found that gain of MM combined with loss FM over time is associated with attenuated FEV1 decline in middle‐aged healthy individuals. In men, loss of FM over time is more closely related with attenuated FEV1 decline than change in MM. Change in body composition over time can be used to identify healthy individuals at high risk of developing chronic lung disease, thus enabling tailored preventive strategies.

## Ethics statement

The study protocol was approved by the Institutional Review Board of Seoul National University Hospital (IRB no. H‐1601‐080‐734) and followed the rules for ethics and data protection which were in accordance with the Declaration of Helsinki. The authors certify that they comply with the ethical guidelines for authorship and publishing of the *Journal of Cachexia, Sarcopenia, and Muscle*.^28^

## Author contributions

P.H.W., P.H.K., and L.S.H. conceived and designed the study. P.H.W., P.H.K., and L.S.H. conducted the primary analysis and data collection. P.H.W., L.S.Y., and K.S.S. interpreted data. P.H.W., P.H.K., and L.S.H. prepared the first draft of the manuscript. All authors reviewed the draft for intellectual content and approved the submission of the final version of the manuscript.

## Conflict of interest

All authors declare that none have conflict of interest related with this article.

## Supporting information


**Figure S1.** Correlations between rate of decline in FEV1 and rate of change in body weight.
**Figure S2.** Correlations between rate of change in body weight and change rate of muscle or fat mass index.
**Table S1.** Distribution of change rate of muscle and fat mass index quartiles.
