# Supplementary material for: Relationships between lung function decline and skeletal muscle and fat mass changes: a longitudinal study in healthy individuals
Source: J Cachexia Sarcopenia Muscle. 2021 Oct 5;12(6):2145–53. doi: 10.1002/jcsm.12821 (PMC8718064; doi:10.1002/jcsm.12821)

**Online Supplementary Material**

Han-Ki Park, MD^1^, So-Hee Lee, MD^2^, Suh-Young Lee, MD, PhD^3^, Sun-Sin Kim, MD, PhD^2^, Heung-Woo Park, MD, PhD^3,4,5^

^1^Department of Internal Medicine, School of Medicine, Kyungpook National University, Daegu, Korea;

^2^Seoul National University Hospital Healthcare System Gangnam Center, Seoul, Republic of Korea;

^3^Department of Internal Medicine, Seoul National University Hospital, Seoul, Republic of Korea;

^4^Institute of Allergy and Clinical Immunology, Seoul National University Medical Research Center, Seoul, Republic of Korea;

^5^Department of Internal Medicine, Seoul National University College of Medicine, Seoul, Republic of Korea

**Supplementary figure legends**

**Figure S1. Correlations between rate of decline in FEV1 and rate of change in body weight**

A. Women

B. Men

**Figure S2. Correlations between rate of change in body weight and change rate of muscle or fat mass index**

A. Rate of change in body weight *vs.* change rate of MMI in women

B. Rate of change in body weight *vs.* change rate of FMI in women

C. Rate of change in body weight *vs.* change rate of MMI in men

D. Rate of change in body weight *vs.* change rate of FMI in men

Correlation is measured by the Pearson’s methods, MMI; Muscle mass index, FMI; Fat mass index

**Table S1. Distribution of change rate of muscle and fat mass index quartiles**

|  |  |  |  |  |  |
| --- | --- | --- | --- | --- | --- |
| **Man** |  |  |  |  |  |
|  | MMI quartile |  |  |  | FMI change rate* |
| FMI quartile | Q1 (n=2346) | Q2 (n=2347) | Q3 (n=2348) | Q4 (n=2347) | ([kg/m^2^]/year) |
| Q1 (n=2346) | 630 (26.85%) | 574 (24.46%) | 547 (23.30%) | 595 (25.35%) | -0.053 (-0.071 ~ -0.024) |
| Q2 (n=2347) | 476 (20.29%) | 591 (25.18%) | 653 (27.81%) | 627 (26.71%) | 0.011 (0.0017 ~ 0.022) |
| Q3 (n=2348) | 520 (22.17%) | 589 (25.10%) | 605 (25.77%) | 634 (27.01%) | 0.053 (0.042 ~ 0.064) |
| Q4 (n=2347) | 720 (30.69%) | 593 (25.27%) | 543 (23.13%) | 491 (20.92%) | 0.12 (0.091 ~ 0.14) |
| MMI change rate*  ([kg/m^2^]/year) | -0.078  (-0.087 ~ -0.064) | -0.046  (-0.051 ~ -0.041) | -0.027  (-0.031 ~ -0.022) | 0.00044  (-0.01 ~ 0.0076) |  |
|  |  |  |  |  |  |
| **Woman** |  |  |  |  |  |
|  | MMI quartile |  |  |  | FMI change rate* |
| FMI quartile | Q1 (n=1521) | Q2 (n=1522) | Q3 (n=1523) | Q4 (n=1522) | ([kg/m^2^]/year) |
| Q1 (n=1521) | 366 (24.06%) | 333 (21.88%) | 356 (23.37%) | 466 (30.62%) | -0.027 (-0.045 ~ 0.0043) |
| Q2 (n=1522) | 325 (21.37%) | 386 (25.26%) | 407 (26.72%) | 404 (26.54%) | 0.042 (0.032 ~ 0.054) |
| Q3 (n=1523) | 382 (25.12%) | 418 (27.46%) | 387 (25.41%) | 336 (22.08%) | 0.087 (0.0075 ~ 0.099) |
| Q4 (n=1522) | 448 (29.45%) | 385 (25.30%) | 373 (24.49%) | 316 (20.76%) | 0.17 (0.13 ~ 0.19) |
| MMI change rate* ([kg/m^2^]/year) | -0.032  (-0.039 ~ -0.021) | -0.0076  (-0.011 ~ -0.0036) | 0.0071  (0.0033 ~ 0.011) | 0.028  (0.019 ~ 0.034) |  |
|  |  |  |  |  |  |

*Mean (95% confidence interval), MMI; Muscle mass index, FMI; Fat mass index, Q; Quartile, n; Number

**Figure S1.**


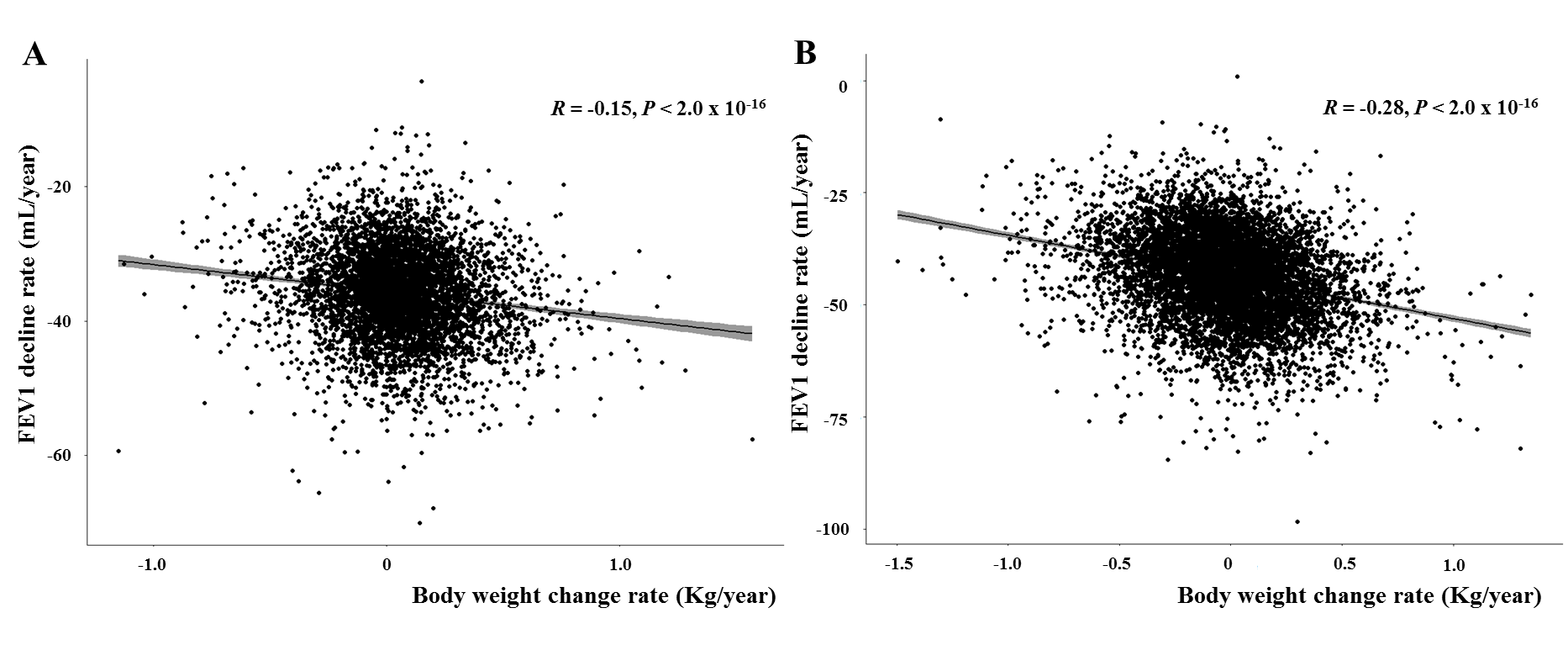


**Figure S2.**


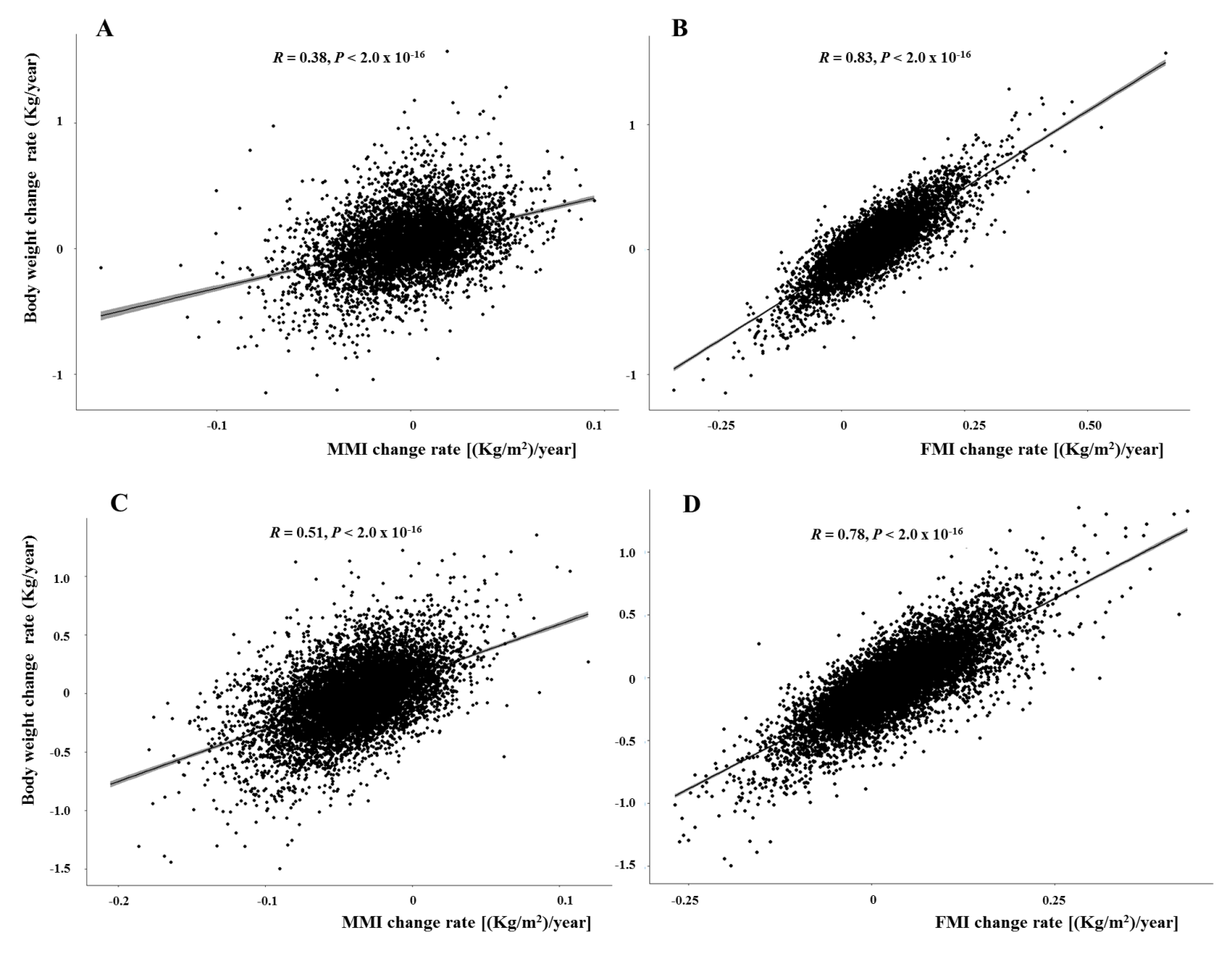

Supplement: Supplementary file 1 — Figure S1. Correlations between rate of decline in FEV1 and rate of change in body weight. Figure S2. Correlations between rate of change in body weight and change rate of muscle or fat mass index. Table S1. Distribution of change rate of muscle and fat mass index quartiles. [file JCSM-12-2145-s001.docx]
